# Commentary: Continuously cumulating meta-analysis and replicability

**DOI:** 10.3389/fpsyg.2015.00565

**Published:** 2015-05-06

**Authors:** Jose D. Perezgonzalez

**Affiliations:** Business School, Massey UniversityPalmerston North, New Zealand

**Keywords:** meta-analysis, CCMA, replication, significance testing, confidence interval, effect size

Braver et al. (2014) article was published by *Perspectives on Psychological Science* as part of a special issue on advancing psychology toward a cumulative science. The article contributes to such advance by proposing using meta-analysis cumulatively, rather than waiting for a long number of replications before running such meta-analysis.

Braver et al.'s article sits well alongside a recent call for reforming psychological methods, under the umbrella of “the new statistics” (Cumming, 2012). As it happens with the latter, the method referred to is not new, only the call to use it is. Indeed, the idea behind a continuously cumulating meta-analysis (CCMA) was already put forward by Rosenthal as far back as 1978 and repeated since (e.g., Rosenthal, 1984, 1991). Yet, the reminder is as relevant today as it has been in the past, more so if we want to get psychology, and our own research within it, at the frontier of science.

I will, however, take this opportunity to comment on an issue which I find contentious: the meaning of the replication used to prove the point. Braver et al. define the criterion for a successful replication as achieving conventional levels of significance. They also identify the typical low power of psychological research as a main culprit for failing to replicate studies. Indeed, they went ahead and simulated two normal populations with a medium effect size mean difference between them, from which they randomly drew 10,000 pairs of underpowered samples. The results they obtained fulfilled power expectations: about 42% of the initial studies, about 41% of the replications, and about 70% of the combined study-replication pairs turned out statistically significant—the latter supposedly supporting the benefits of CCMA over the uncombined studies.

What the authors fail to notice, however, is that the meaning of replication differs depending on the data testing approach used: Fisher's approach is not the same than Neyman–Pearson's (Neyman, 1942, 1955; Fisher, 1955, 1973; MacDonald, 2002; Gigerenzer, 2004; Hubbard, 2004; Louçã, 2008; Perezgonzalez, 2015a). Neyman and Pearson's approach (1933) is based on repeated sampling from the same population while keeping an eye on power, which is Braver et al.'s simulation setting (Neyman and Pearson, 1933). However, under this approach a successful replication reduces to a count of significant results in the long run, which translates to about 80% of significant replications when power is 0.8, or to about 41% when power is 0.41. Albeit not intentionally pursued, this is what Braver et al.'s Table 1 shows (power lines 1 and 2, and criteria 1, 2, and 4—combining studies is not expected under Neyman–Pearson's approach but, given the nature of the simulation, such combination can be taken as a third set of studies that uses larger sample sizes and, thus, more power; criteria 5–10 can be considered punctilious studies under criterion 4). That is, Braver et al.'s power results effectively replicate the population effect size the authors chose for their simulation.

On the other hand, the 10,000 runs of study-replication pairs address replication under a different testing approach, that of Fisher's, arguably the default one in today's research (Spielman, 1978; Johnstone, 1986; Cortina and Dunlap, 1997; Hubbard, 2004; Perezgonzalez, 2015b). Under Fisher's approach (1954), power has no inherent meaning—a larger sample size is more sensitive to a departure from the null hypothesis and, thus, preferable, but the power of the test is of no relevance. There is not knowing (or guessing) the true population effect size beforehand, either, in which case meta-analysis helps to approximate better the unknown effect size, exactly what Braver et al.'s Table 2 illustrates. It is under this approach that accumulating studies works, as a way of increasing our knowledge further—something that Fisher (1954) had already suggested. This is also the approach under which Rosenthal presented his techniques for meta-analysis—indeed, he did not contemplate power in 1978 or 1984, and his mentioning it in 1991 seems to be rather marginal to the techniques themselves.

There are other ways of carrying out replications, though, ways more attuned to the “new statistics”—which is to say, ways already discussed by Rosenthal (1978). One of these is to attend to the effect sizes of studies and replications, to better know what we want to know (Cohen, 1994) instead of merely making dichotomous decisions based on significance (Rosenthal, 1991). Another way is to attend to the confidence intervals of studies and replications, as Cumming (2012) suggests.

In summary, Braver et al.'s call for CCMA is a worthy one, even if their simulation confused the meaning of replication under different testing approaches. One thing left to do for this call to have better chances of succeeding is to make CCMA easier to implement. For such purpose, the interested researcher has a suite of readily available meta-analysis computer applications for Microsoft's Excel, such as ESCI (http://www.latrobe.edu.au/psy/research/cognitive-and-developmental-psychology/esci) and MIX (http://www.meta-analysis-made-easy.com), and standalone computer programs such as RevMan (http://tech.cochrane.org/revman) and CMA (http://www.meta-analysis.com)—for more resources see also https://www.researchgate.net/post/Which_meta-analysis_software_is_easy_to_use/1.

## Conflict of interest statement

The author declares that the research was conducted in the absence of any commercial or financial relationships that could be construed as a potential conflict of interest.
